# The Age of the Earth

**Published:** 1893-02

**Authors:** 


### THE AGE OF THE EARTH.

Geologists have ascertained that the rate at which erosion takes place can be measured; by applying their scale to the sedimentary rocks they have formed a hypothesis as to the time which has elapsed since erosion began.

The stratified rocks attain an average thickness of 100,000 feet. The material of which they consist was all washed down from high planes, deposited and left to stratify. By the inspection of river banks it is found that in places the surface of the land which has been carried down as sediment in rivers has been reduced at the rate of a foot in 730 years, while in other places, where the land was more stubborn or less flexible, it had taken 6,800 years to lower the surface one foot. The deposit must be equal to the denudation.

We find that while some of the sedimentary rocks have grown a foot in 730 years, others have taken 6,800 years to rise that height. Thus the period of time that was required to build up 100,000 feet of sedimentary rock has varied according to locality from 73,000,000 years to 680,000,000 years. It follows that the active work of creation lasted for a cycle intermediate between these two figures. The cycle varied with endless succession of periods of disturbance by volcanic force and glacial action, and the frequent submersion of dry land, alternating with the emerging of continents out of the seas. These may have retarded the growth of sedimentary rocks, but they cannot have accelerated it.

A study of fossils teaches the steady uniformity with which the work of creation proceeded. Since man began to observe there has been no change in the forms of animal and vegetable life. A few species have disappeared—not one new species has been evolved. Not only do we find the fauna and flora of ancient Egypt as depicted on monuments which are probably 8,000 or 10,000 years old, identical with those which are found in that country to-day, but shells which inhabited our seas before the ice age and grew in an ocean whose bed overlay the Rocky Mountains are precisely the same species that are found in the Bay of Monterey and the waters of the Chesapeake. It is evident that there has been no essential change in the conditions of life since these animals and these vegetables were first created, yet how vast the shortest period that divides us from that remote epoch !

